# Differences in the healthcare needs of older adults attending primary health centers in urban and rural areas of Taiwan

**DOI:** 10.1186/s12875-023-02174-7

**Published:** 2023-10-19

**Authors:** Chen-I Shih, Hui-Fei Yang, Shu-Li Chia, Tang-Kuei Lin, Sheng-Yu Fan

**Affiliations:** 1 Department of Community Health, Ditmanson Medical Foundation Chia-Yi Christian Hospital, Chia-Yi, Taiwan; 2 Health Promotion Administration, Ministry of Health and Welfare, Taipei, Taiwan; 3 East District Primary Health Center, Chia-Yi, Taiwan; 4 Institute of Gerontology, College of Medicine, National Cheng Kung University, No.1, University Road, Tainan City, 701 Taiwan

**Keywords:** Age-friendly, Health care services, Primary health centers, Rural-urban difference

## Abstract

**Purpose:**

As the older adult population in Taiwan continues to increase, primary health centers (PHCs) play a crucial role in geriatric care. This study explored the differences in the PHC experiences and usage needs of older adults in urban versus rural areas.

**Methods:**

A qualitative study was conducted. Twenty-one older adults were recruited from PHCs in northern, central, southern, and eastern Taiwan. Semistructured interviews were used to collect data, and the interview guidelines included their reasons for visiting PHCs, the health-care services they received, their evaluation of the services, and the advantages and disadvantages of these centers. The data were analyzed using thematic content analysis.

**Results:**

The PHC usage needs of older adults in urban areas differ from those of older adults in rural areas in the following 3 aspects: (1) Medical services: older adults in rural areas demand clinics specializing in various medical domains, mobile and home medical care, and case management, whereas those in urban areas demand mobile health examinations. (2) Environment and transportation: older adults in rural areas demand diverse medical equipment, shuttle services, and accessible facilities, whereas those in urban areas demand recreational facilities. (3) Active aging: older adults in rural areas demand health education courses, and those in urban areas demand diverse senior citizen courses as well as opportunities to volunteer and build interpersonal relationships.

**Conclusion:**

The older adults in urban and rural areas had different PHC usage needs. The older adults in rural areas generally focused on medical care and transportation services in PHCs, whereas those in urban areas generally focused on health promotion as a means of social participation and active aging.

## Introduction

Many countries [1], including Taiwan, have aging populations [2]. Following an increase in the older adult population in Taiwan, the percentage of older adults using National Health Insurance resources has increased continually [3], with chronic diseases being the primary factor contributing to said usage [4]. According to a national health survey, 86.3% of older adults have been diagnosed with at least one chronic disease [2]. Preventing chronic diseases, promoting older adults’ health, and maintaining older adults’ physical function are key goals in society [5].

The purposes of primary health centers (PHCs) are to promote older adults’ health, prevent and monitor their diseases, and reduce their physical disabilities [6]. PHCs provide older adults with health education, risk assessments, health screening, and primary care; when further medical care is required for older adults, they are referred to receive appropriate medical services [7]. Additionally, PHCs integrate community resources to promote older adults’ social participation and help them live independently [7]. This includes shuttle services and access to facilities, including those used for community service projects [8].

The urban–rural gap, which has long been a prominent problem worldwide, is caused by the lower socioeconomic status and poorer health behaviors of rural populations compared with urban populations [9]. It is also a consequence of the unequal distribution of medical resources; rural areas have fewer medical personnel, lower medical coverage, and a longer distance to medical centers than urban areas [9]. Taiwan has many mountainous areas with uneven distribution of population and healthcare resources. In 1995, Taiwan started promoting its National Health Insurance program to remove financial barriers to medical care, but an urban–rural gap remains [9]. Chronic disease management is required in rural PHCs because it can reduce the number of hospitalizations and mitigate problems in the community associated with chronic diseases [10]. With the increase of aging population, PHCs in Taiwan have to take responsibility to provide primary care for older adults in different areas.

Older adults in urban areas have more medical resources than those in rural areas do [11]; they are also more willing to participate in social activities [12] and visit PHCs more frequently [13]. Community environment is closely associated with older adults’ health behaviors, the comfort, safety, and accessibility of related facilities affect older adults’ intentions to participate in community activities. Therefore, community building must be planned in consideration of older adults’ needs to promote their physical, psychological, and social health [13]. The present study explored the differences in the PHC experiences and usage needs between older adults in urban and in rural areas in Taiwan. The results can be used to establish primary health-care policies that meet the requirements of older adults in both types of areas.

## Methods

### Participants

The participants were Han Taiwanese aged 65 years or older and indigenous Taiwanese aged 55 years or older. According to the Senior Citizens Welfare Act [14], indigenous people aged 55 years or older qualify as older adults. They could communicate in Mandarin or Taiwanese. Purposive sampling was conducted for age, sex, and residential area. The participants were recruited from PHCs in northern, central, southern, and eastern Taiwan. Based on the criteria of the Ministry of the Interior of Taiwan, urban areas are defined as a population over 200,000 in main cities or areas, and rural areas are defined as a population density lower than one-fifth of the national average population density that 129 inhabitants per square kilometer. Older adults who had cognitive problems, were unable to speak clearly and fluently, or refused to be interviewed were excluded.

### Study design and data collection

This study was approved by the institutional review board of the regional hospital (IRB number: CYCH-IRB2019097), and informed consent was obtained before the semistructured interviews were conducted for data collection. The interview guideline was as follows: (1) primary reasons for visiting PHCs and the health care services used; (2) perceived advantages and disadvantages of older adult care provided by the PHCs; (3) perceived advantages and disadvantages of PHC environments; and (4) suggestions for improving the older adult care provided by the PHCs.

The interview guideline was reviewed and modified by a professor of public health, a professor of sociology, and a senior PHC director before the interviews commenced. The demographic information of the participants was also recorded, including regions, the characteristics and services of the PHCs visited, sex, age, ethnicity, frequency of visits, and reasons for visiting PHCs.

### Data analysis

The interviews were audio recorded, transcribed verbatim, and subjected to a thematic content analysis [15], which involved the following steps: (1) Familiarization with the data: Read the verbatim script repeatedly to identify relevant information. (2) Generation of initial codes and building of a framework: Mark the themes mentioned by the interviewees, give them titles as codes, and categorize and organize them. For example, the response of an interviewee expressing that they enjoyed having frequent interactions with people was coded as one related to interpersonal relationships. All instances of interview content coded as related to interpersonal relationships were grouped together. (3) Construction of themes: After the code framework is established, group the codes with meanings to establish themes, and construct stories using the themes. For example, the three codes of educational courses, interpersonal relationships, and volunteering contributions were combined under the theme of “active aging to promote the physical and psychological functioning of older adults.” (4) Review of themes: Examine each theme and code it for fitness; coding should proceed based on whether each theme forms a reasonable and complete story with relevant codes. (5) Define and name the themes: Examine the meaning of each theme and give it a clear name.

The rigor of this qualitative study was verified through the following steps: (1) The researchers utilized their extensive experience in qualitative research and the implementation of age-friendly programs to establish mutual trust and record the interviews to ensure data authenticity. (2) The interviews were transcribed within 72 h after their interview to prevent data loss caused by memory ambiguity. Data was collected until data saturation that no new information was found in data analysis. (3) The researchers regularly clarified the data with the participants and verified that the transcripts corresponded to the research questions to ensure the robustness of the data. (4) The first author (CIS) conducted the interviews and a research assistant also joined to record the process. The interviewer and research assistants received eight hours trainings about the interview guidelines and the skills related to observing and interviewing older adults, ensuring data consistency and repeatability. The data were categorized and induced using Atlas.ti 6.0 [16].

## Results

In total, 27 participants were recruited and only 21 were included. Six participants were excluded because they did not meet the inclusion criteria. Ten participants were from urban areas, and 11 participants were from rural areas. Seven of these participants were men. Seven of the participants were indigenous Taiwanese. Regarding the reasons for PHC visits, 11 and 8 of the participants visited PHCs for internal medicine and vaccination, respectively (See Table 1).

**Table 1:** Demographic characteristics of the participants

	Urban PHC (n = 10)	Rural PHC (n = 11)
Sex		
Male	2	5
Female	8	6
Age		
60–69 years old	7	4
70–79 years old	3	5
80–89 years old	0	2
Ethnicity		
Han Taiwanese	4	11
Indigenous Taiwanese	7	0
Frequency of visits		
Every week	6	7
Every month	1	4
Every three months	3	1

Three themes were revealed in relation to the urban–rural gap and participants’ PHC needs, including medical services, environment and transportation, and active aging (See Table 2).

**Table 2:** Themes and subthemes

Theme	Subtheme	Urban (Numbers of coding)	Rural (Numbers of coding)
Medical services	Outpatient care	1. Medical classification (20)	1. Visit PHCs for all diseases (12) 2. Insufficient medical resources (31)
	Mobile and home medical care	1. Mobile health examination (12)	1. Mobile medical care (23) 2. Home medical care (6)
	Case management	1. Not every older adult receives individual notifications about PHC activities (9)	1. Sufficient continuity of care for each older adult (19)
Environment and transportation	Medical facilities	1. Sphygmomanometer data recorded in the cloud (1)	1. Various medical testing instruments (6)
	Shuttle services	1. Convenient for autonomous travel (16)	1. Shuttle buses available (7) 2. Insufficient shuttle buses (6)
	Age-friendly environment	1. Recreational facilities (5)	1. Insufficient accessibility (4) 2. Insufficient software to replace hardware (6)
Active Aging	Educational courses to promote active aging	1. Policy promotion about active aging (2) 2. Exercise and health (30) 3. Spiritual growth (16) 4. Hobbies and leisure activities (20)	1. Exercise and health (15) 2. Hobbies and leisure activities (1)
	Interpersonal relationships	1. Interpersonal connections through courses (17)	1. Close relationships among villagers (3)
	Volunteering contributions	1. Volunteer roles (11) 2. What it means to be a volunteer (24)	N/A

### Theme 1. Medical services

The medical services in urban and rural areas differed in their outpatient clinics, mobile medical care and home care, and care management.

#### Outpatient medical care

Various levels of medical institutions are available in cities, older adults with severe medical conditions visit hospitals, and only those with mild or stabilized chronic diseases visit PHCs. However, because medical resources are insufficient in rural areas, older adults with medical needs in such areas usually seek help from local PHCs first. These PHCs must provide interdisciplinary medical care to fulfill the needs of older adults. Currently, retaining medical personnel in rural PHCs remains difficult, and older adults in rural areas have frequently complained about a lack of doctors.

##### Case 20

*Blood pressure–lowering drugs can be accessed in PHCs. Only severe diseases must be treated in big hospitals. (Urban)*

##### Case 7

*It’s inconvenient to get medical care because rural PHCs are short on doctors. Those doctors are transferred after merely a few months of stay, and we have to wait a long time to get a new doctor. (Rural)*

#### Mobile and home medical care

Urban areas have abundant medical resources. Although older adults in these areas rarely use mobile and home medical care provided by PHCs, PHCs provide mobile health examinations to older adults living in communities, making medical access easier. The medical services provided by urban PHCs were affirmed by older adults. By contrast, rural areas have insufficient medical resources, and older adults in these areas rely on mobile and home medical care provided by PHCs. Those who live far away from PHCs pay regular return visits, and PHCs perform mobile medical care in communities. Home medical care is praised by older adults in rural areas because it has vastly improved their medical access.

##### Case 2

*It’s convenient to join the PHC community activities nearby. I have received health examinations, blood sugar tests, blood pressure tests, pap smears, and mammograms. (Urban)*

##### Case 9

*The PHC staff are very thoughtful in giving mobile medical care. They come once every week, and I would go see the doctor every week. (Rural)*

#### Case management

Rural PHCs offer more individualized health care to older adults than urban PHCs, including screening patients, establishing customized health service plans, and regular tracking and monitoring. Older adults in rural areas receive more continuous care from PHCs than those in urban areas do. PHCs in urban areas provide fewer medical services than do rural PHCs; they only offer group-based health service plans and do not provide sufficient individualized health services to older adults within their localities.

##### Case 11

*The PHC would come to our village and see if there are any patients, and patients with severe conditions are referred immediately. Everything is well taken care of this way. (Rural)*

##### Case 1

*I have learned about PHC activities only when I go there, I can participate in these activities if I have phone or SMS notifications. (Urban)*

### Theme 2. Environment and transportation

Older adults in urban and rural areas have different needs with respect to medical facilities, shuttle buses, and the establishment of an age-friendly environment.

#### Medical facilities

Rural PHCs have many types of advanced medical examination equipment that urban PHCs do not possess. Because of the medical resource insufficiency in rural areas, diverse medical facilities are required for rural PHCs to provide instant screening services to older adults. However, urban PHCs use cloud devices, such as cloud-based sphygmomanometers, to enable older adults to monitor their own health autonomously via the cloud network. Older adults are unfamiliar with cloud products and rely on assistance from service personnel to use them effectively.

##### Case 11

*Our PHC provides screening devices (…) Currently, I know they have electrocardiograms. They also screened my liver once. (Rural)*

##### Case 3

*My health condition is recorded on the cloud, and on the computer is a sphygmomanometer, but I didn’t know how to use it. A service provider later came and helped me to use it. (Urban)*

#### Shuttle services

Older adults in urban areas live in proximity to PHCs, and few of such adults require shuttle services to access these PHCs. By contrast, older adults in rural areas require constant shuttle services and have expressed that transportation is a major problem they face. They have praised PHCs that provide shuttle buses. However, not all rural areas provide shuttle services, and some older adults have difficulty accessing such services.

##### Case 11

*Transportation problems are critical to look at (…) Our shuttle services are good. We have one shuttle bus to the PHC and one to the hospital. (Rural)*

#### Age-friendly environment

Most older adults require accessible facilities, and both urban and rural PHCs have their accessible facilities continually maintained and repaired. In addition, older adults require recreational facilities to ensure active aging, such as exercise, social, and leisure facilities. Urban PHCs have a larger number of recreational facilities than rural PHCs, and older adults in urban areas are satisfied with these facilities because they have improved their interpersonal relationships. By contrast, older adults in rural areas have been less satisfied with the accessible facilities in rural PHCs. Rural PHCs have improved the shortcomings of hardware facilities through many alternatives such as voluntary support for older adults with difficulty moving.

##### Case 20

*The PHC’s hardware facilities are good. The karaoke devices are easy to use, and I’m happy. I can have fun with everyone. (Urban)*

##### Case 7

*Speaking of accessible facilities, when I ride up to the PHC in my electric scooter, the corner is too narrow. The stair treads are also too narrow. (Rural)*

### Theme 3. Active aging

Urban and rural PHCs have different approaches to promoting active aging, with approaches including educational courses, interpersonal relationships, and volunteer services.

#### Educational courses for active aging

Urban PHCs provide diverse courses to promote older adults’ lifelong learning, physical and mental health, and social participation. Educational courses are provided on topics such as medicine, exercise, cognitive function, and leisure activities; older adults can choose courses that suit their requirements. By contrast, older adults in rural areas have fewer course choices because of resource limitations. They can only participate in exercise courses when their PHCs bring instructors to their communities during mobile medical care services. The courses on leisure activities from rural PHCs are also not as diverse as those from urban PHCs and are mainly led by older adults with specific talents in communities.

##### Case 20

*The PHC would host exercise, medical, or dementia-related courses (…) They also provide courses on Taiwanese operas, tabletop games, and handicrafts (…) I would go to the courses every day. (Urban)*

##### Case 9

*I have been in exercise courses for older adults. When our PHC gives mobile medical care, an instructor would come along and teach us aerobics. (Rural)*

#### Interpersonal relationships

Urban and rural PHCs differ in their approaches to promoting older adults’ interpersonal relationships. Urban PHCs provide older adults with educational courses to promote active aging, and urban PHCs are hubs for social interactions. Therefore, older adults in urban areas make friends easily at PHC courses. By contrast, older adults in rural areas do not rely on PHCs to form interpersonal connections. They create their own social circles and invite others to visit PHCs together, displaying strong social cohesion.

##### Case 20

*I am happy when I make friends in PHC courses because I like interacting with people. The people in the PHC get along well, like sisters. (Urban)*

##### Case 13

*We contact each other when we haven’t seen each other for one week. We all cherish our emotional connections as older adults in the community. (Rural)*

#### Volunteering contributions

Urban PHCs provide older adults with opportunities to volunteer, and these older adults are often highly enthusiastic about volunteering. Their roles in PHCs include helping patients measure their blood pressure and body temperature, fill in data, and see a doctor. These older adults volunteer to help others and give back to society, earning life satisfaction and happiness. By contrast, older adults in rural areas have not mentioned the volunteer service vacancies from their PHCs, nor did they mention having any intention to volunteer.

##### Case 18

*In the PHC, we accompanied patients in clinical visits, measured their blood pressure and body temperature, and guided them to see a doctor after they made appointments (…) I’m old, and I help others when I can. (…) I want to give back to society (…) I’m satisfied as long as I can volunteer (…) Volunteering makes me happy. (Urban)*

## Discussion

This qualitative study explored the differences in older adults’ use of PHC services related to medical resources, the environment and transportation, and active aging. Older adults in rural areas have a greater need for diverse medical facilities, interdisciplinary diagnostic and treatment options, and mobile and home medical care being offered by PHCs than those in urban areas do. By contrast, older adults in urban areas have a greater need for educational courses on physical and psychological health and having opportunities to volunteer, which can fulfill their social participation needs.

An urban–rural gap exists in Taiwan in terms of medical services [9]. Rural areas lack sufficient medical resources, and PHCs may be the only available places for older adults in these areas to access medical services. Similar to Greece [17] and Indonesia [18], older adults in rural areas have disadvantage in using PHCs service. Therefore, interdisciplinary medical care is particularly important in rural PHCs [19], and individualized health management is necessary for effective chronic disease control and health promotion [20]. Moreover, rural areas are geographically disadvantaged and older adults have difficulty moving, and rural PHCs must provide mobile and home medical care to improve medical service accessibility [21]. Older adults with chronic diseases receive case management in rural PHCs and in outpatient clinics through screening, care programs, condition evaluation, and disease monitoring [22]. Although rural PHCs currently provide older adults with various services, difficulty in retaining doctors continues to encumber these PHCs. In response, the central government strengthens rural medical personnel training and provides retention bonuses to mitigate medical workforce shortages in rural areas [23].

Older adults in urban areas are not reliant on PHCs for medical access. Older adults in such areas generally wish that PHCs would provide mobile health examinations in their communities and provide individualized notifications related to health-care activities. In response, the central government promotes community disease screening; specifically, PHCs across Taiwan are tasked with providing mobile health examinations at specific locations in each community, offering older adults easy access to health examinations [24]. Because the nurse–patient ratio in urban areas is low [25], urban PHCs tend to focus on group-based medical care and provide resources and referral services to groups of people with similar medical needs, thereby improving group health [26].

The geographic conditions of rural areas lead older adults in such areas to have specific needs related to medical facilities and transportation. Older adults in rural areas have a greater need for public transportation, medical shuttle services, and telemedicine than those in urban areas do [27]. This study revealed that older adults in rural areas expect their PHCs to provide diverse medical facilities, shuttle buses, and accessible facilities, whereas those in urban areas expect favorable recreational facilities. Therefore, the central government has established medical facilities and 5G online clinics in rural PHCs. Additionally, medical imaging services such as x-ray, ultrasounds, and electrocardiograms have been implemented or upgraded. Older adults in rural areas are offered shuttle services. An integrated delivery system is promoted to facilitate collaboration between hospitals and rural PHCs in providing local clinics and emergency care through medical vehicles and first-aid stations [28]. Age-friendly environments are renovated; specifically, accessible and recreational facilities are refurbished according to the needs of the attendees of urban or rural PHCs [29]. Rural PHCs have provided alternative measures to enhance older adults’ access to medical treatment, such as support for those with limited mobility.

Taiwan government promotes the policy of age-friendly PHCs and addresses not only disease prevention but also health promotion in PHCs. PHCs are responsible for the promotion of healthy and active aging, which involves older adults optimizing their physical health, psychological cognition, emotional regulation, and social function [30]. Urban and rural PHCs employ different approaches to promoting active aging. This study indicated that rural PHCs have less diverse and frequent active aging courses than urban PHCs. The learning resources and formal classrooms for older adults in rural areas have been scarcer than have those in urban areas; when older adults in rural areas encounter problems, they usually ask for the opinions of their families and friends. Because the close bonds in rural communities are conducive to building trust and positive environments for discussion, autonomous learning has been the most preferred learning model by older adults in many countries [31]. The central government plans to design a remote technology platform for experts to provide online learning services to older adults, which could mitigate the geographic restrictions to learning resource access [32].

PHCs in rural areas take medical services as the priority and may not have enough resources to provide various kinds of courses. On the other hand, urban areas have abundant medical resources. PHCs in urban areas have less demands on medical services and have more resources to provide health promotion courses. Therefore, such PHCs can focus on promoting active aging through educational courses in which older adults can form interpersonal connections. Consequently, older adults in urban areas more frequently participate in social activities [12]. Older adults in urban areas exhibit strong intentions to participate in the courses and volunteer services provide by PHCs. Urban PHCs offer many types of courses to older adults as well as a learning environment conducive to strengthening interpersonal relationships, enabling older adults to support each other in their exchanges, discuss their opinions in a safe environment, and enrich their own learning experience [33]. Older adults’ psychological health can also be improved through social connections in courses, thereby promoting active aging [19]. Autonomous participation in volunteer services satisfies older adults’ psychological needs and improves their life satisfaction [34]. Accordingly, urban PHCs have substantially contributed greatly to the active aging of older adults in urban areas.

The present study focused on the subjective experiences of older adults who had used PHCs. Because of geographical differences and the unequal distribution of medical resources, older adults in urban and rural areas have different needs for PHCs. Urban PHCs focus on health screening and the courses of active aging, while as rural PHCs take medical care and disease prevention as priority, as well as the needs of transportation for accessing services. Medical care is one of the most important needs of older adults, and those in rural areas may have unmet needs. Therefore, Taiwan government uses some strategies, including budget for medical staff, shuttle buses, and telecommunication, to solve the problems.

Some limitations of this study should be acknowledged. First, only qualitative data were collected, with these data being collected from subjective interviews. In future studies, other forms of data, such as nonparticipatory observations, medical case analyses, National Health Insurance data, and health examination records can be employed to strengthen data credibility and integrity. Second, the participants were recruited through purposive sampling and, therefore, may be inadequately representative. Quantitative studies with random sampling should be conducted to compare older adults in urban areas with those in rural areas based on their requirements when using PHCs, increasing the generalizability of relevant research results.

## Conclusion

Urban and rural PHCs differ in their missions for improving older adults’ health. Because of the urban–rural gap in medical resources, rural PHCs focus on medical care; these PHCs must provide clinics that specialize in various medical domains; diverse health examination equipment; mobile medical care services; and shuttle services to elevate the accessibility of medical services for older adults. Older adults in urban areas visited PHCs less often because of the diverse medical service options available to them. Consequently, urban PHCs focus on providing older adults with health examinations and promoting active aging to improve older adults’ health.

## Data Availability

The data generated and analysed during the current study are not publicly available due to participant consent but are available from the corresponding author on reasonable request.
